# Gene Polymorphisms of *TLR4* and *TLR9* and *Haemophilus influenzae* Meningitis in Angolan Children

**DOI:** 10.3390/genes11091099

**Published:** 2020-09-21

**Authors:** Elina Tenhu, Johanna Teräsjärvi, Manuel Leite Cruzeiro, Okko Savonius, Emilie Rugemalira, Irmeli Roine, Qiushui He, Tuula Pelkonen

**Affiliations:** 1 Institute of Biomedicine, Research Center of Infections and Immunity, University of Turku, 20520 Turku, Finland; elpate@utu.fi (E.T.); johter@utu.fi (J.T.); 2 Hospital Pediátrico David Bernardino, Luanda 00000, Angola; mcruzeiro1@gmail.com (M.L.C.); tuulapelkonen@hotmail.com (T.P.); 3 Children’s Hospital, Pediatric Research Center, University of Helsinki, Helsinki University Hospital, 00029 Helsinki, Finland; okko.savonius@helsinki.fi (O.S.); emilie.rugemalira@helsinki.fi (E.R.); 4 Faculty of Medicine, University Diego Portales, Santiago 8370109, Chile; irmeli.roine@gmail.com; 5 Department of Medical Microbiology, Capital Medical University, Beijing 100069, China

**Keywords:** TLR4, TLR9, gene polymorphisms, meningitis, *Haemophilus influenzae*, HRMA, children, Angola

## Abstract

Bacterial meningitis (BM) is a severe disease caused by various bacterial pathogens. Toll-like receptors (TLRs) protect humans from invading pathogens. In this study, we determined whether single nucleotide polymorphisms (SNPs) of *TLR4* and *TLR9* are associated with susceptibility to and outcome of BM in Angolan children. Samples were taken from 241 patients and 265 age-matched ethnic controls. The SNPs *TLR4* rs4986790 (896A > G) and *TLR9* rs187084 (−1486T > C) were determined by high-resolution melting analysis (HRMA). The frequency of variant genotypes in *TLR4* was significantly higher in patients with *Haemophilus influenzae* meningitis than controls (odds ratio (OR), 2.5; 95% confidence interval (CI), 1.2–5.4; *p* = 0.021), whereas the frequency of variant genotypes in *TLR9* was significantly lower in patients with *H. influenzae* meningitis than controls (OR, 0.4; 95% CI, 0.2–0.9; *p* = 0.036). No such differences were found with other causative pathogens, such as *Streptococcus pneumoniae* and *Neisseria meningitidis*. At the time of discharge, patients with meningitis caused by Gram-negative bacteria who were carriers of variant *TLR4* genotypes had a higher risk of ataxia (OR, 12.91; 95% CI, 1.52–109.80; *p* = 0.019) and other neurological sequelae (OR, 11.85; 95% CI, 1.07–131.49; *p* = 0.044) than those with the wild-type *TLR4* genotype. Our study suggests an association between *H. influenzae* meningitis and genetic variation between *TLR4* and *TLR9* in Angolan children.

## 1. Introduction

Bacterial meningitis (BM) is a life-threatening infectious disease. Even with early diagnosis and proper treatment, the fatality rate of BM ranges from 5% to 10% in developed countries and up to 50% in low-and middle-income countries. It is estimated that approximately 1–2 million cases of BM occur a year [1], with neonates particularly susceptible. Survivors of meningitis are at an increased risk of neurological sequelae, such as learning and behavioral disorders, deafness, paresis, and severe encephalopathy [2]. The most common prerequisite for BM is nasopharyngeal colonization and bacteremia, where bacteria can spread from blood to cerebrospinal fluid (CSF). The pathogens can infect the central nervous system (CNS) by crossing the blood–CSF barrier, invading the CSF directly through a skull fracture, or from a focal infection (e.g., the nasal sinuses) to the subarachnoid space [2,3,4].

BM is caused by various bacteria such as *Streptococcus pneumoniae*, *Neisseria meningitidis*, and *Haemophilus influenzae*. *H. influenzae* is still one of the most common causes of BM in non-industrialized countries. This is due to the lack of effective vaccinations, which have virtually eliminated *H. influenzae* type b meningitis in industrialized countries [5]. Both *H. influenzae* and *N. meningitidis* are Gram-negative bacteria carried in the nasopharynx. *H. influenzae* is a common causative agent of BM in young children [6,7]. Around 10% of people, mostly school-aged children, carry *N. meningitidis* in their nasopharynx, and *N. meningitidis* is the main cause of BM in children and young adults. *N. meningitidis* causes epidemics, particularly in sub-Saharan Africa (the “meningitis belt”). Pneumococcal nasopharyngeal carriage is common in the general population and affects people of all ages. *S. pneumoniae* causes the most severe disease in both young and old people [2,8,9,10].

Routine immunizations in Angola include the pentavalent vaccine (i.e., diphtheria–tetanus–pertussis (DTP), hepatitis B, and *H. influenzae* type b), which was introduced in 2006, and the 13-valent pneumococcal vaccine (PCV13), which was introduced in 2013 [11]. Both are administered at 2, 4, and 6 months of age. The current estimates of the coverage of three doses of DTP vaccine in children in Angola range from 48% to 84% [12,13], while estimates for PCV13 vaccination rates range from 59% to 82% [11,12].

The number of children with BM has been decreasing in the Paediatric Hospital of Luanda following the introduction of routine childhood pentavalent vaccination in 2006. In particular, the number of BM cases due to *H. influenzae* type b has dropped by 86% in the Paediatric Hospital [14]. In 2004, before the use of PCV13 in Angola, there were 93 cases of pneumococcal meningitis identified in children in this hospital; however, our one-year study found 43 cases of pneumococcal meningitis, indicating a large decline of BM caused by *S. pneumoniae* in the post-PCV13 period [5].

Pathogen-sensing Toll-like receptors (TLRs) are key components of innate and adaptive immunity. To date, ten TLRs have been identified in humans (i.e., TLR1 to TLR10). TLR4 is expressed on the cell surface and recognizes the lipopolysaccharides (LPSs) of *H. influenzae* and *N. meningitidis,* as well as pneumolysin produced by *S. pneumoniae* [15]. TLR4 requires the adaptor protein MD2 to bind to the lipophilic part of LPSs, which activates intracellular signaling and leads to a host immune response to invading pathogens [16,17,18,19]. TLR9 is localized in the endosomal compartments of cells. It is specialized in detecting bacterial un-methylated single-stranded DNA—more specifically, unmethylated cytosine–guanosine-containing oligonucleotide (CpG-DNA). TLR9 requires the DNA-degrading enzyme DNase to sense single-stranded DNA [16,18,20]. It has been observed that *S. pneumoniae*, *H. influenzae*, and *N. meningitidis* can activate species-specific patterns of TLR4 and TLR9 [21].

Genetic factors are major determinants of human susceptibility to infectious diseases. A common type of genetic variation is single-nucleotide polymorphisms (SNPs), which, in immune genes such as *TLR*s, are involved in the susceptibility, severity, and outcome of different bacterial and viral infections [22]. Two well-studied functional SNPs of *TLR4* (rs4986790) and *TLR9* (rs187084) were included in this study. The polymorphism of *TLR4* Asp299Gly impairs TLR4-mediated LPS signaling and results in decreased LPS-stimulated NFκB activity [23]. In our previous study, we found that this polymorphism increases the risk of nasopharyngeal colonization of *H. influenzae* and *Moraxella catarrhalis* in healthy Finnish children [24]. This polymorphism is also associated with septic shock [25], respiratory syncytial virus (RSV) bronchiolitis [26], sepsis [27], and urinary tract infection (Table 1). However, previous studies have also shown that the *TLR4* polymorphism does not increase the risk of meningococcal or pneumococcal diseases such meningitis or sepsis [28,29,30]. The polymorphism *TLR9* rs187084 is associated with streptococcal infections and tuberculosis, as well as autoimmune disorders and cancers [31,32,33].

There are other *TLR4* and *TLR9* SNPs that are associated with BM. In one study, it was found that the carriage of one or both mutant alleles in *TLR4* 896A > G and *TLR9*-1237 increases the risk of hearing loss in BM survivors. Another study found that BM patients who carried the minor allele T of *TLR9* (rs352140) developed seizures more often than those without. It has also been reported that there is an association between *TLR9* SNPs and susceptibility to BM, specifically meningococcal meningitis. Although an association between the *TLR9* polymorphism rs187084 and BM has not yet been described, previous studies with other *TLR9* polymorphisms indicate that this polymorphism may also play a role in BM susceptibility or outcome [3,34,35].

Thus, our goal was to identify the functional gene polymorphisms of *TLR4* and *TLR9* that affect the susceptibility to and severity and outcomes of BM in Angolan children.

## 2. Materials and Methods

### 2.1. Subjects

This study was a part of two prospective randomized clinical trials of children with BM in the Luanda Children’s Hospital (Hospital Pediátrico David Bernardino) Luanda, Angola. The studies were approved by the Luanda Children’s Hospital ethics committee. We assessed all children aged 2 months to 15 years presenting with signs suggestive of BM. A child was enrolled if their CSF appeared cloudy, was positive by Gram staining, or showed >50 leukocytes/mm^3^. The exclusion criteria included previous neurological abnormalities or hearing impairment, immunosuppression except for HIV infection, active tuberculosis, known hepatic disease, or pre-treatment with more than one dose of parenteral antibiotic. The patients were included in the study after informed consent was provided by their guardians [36,37]. Oral informed consent was also obtained from the guardians of the controls.

The study cohort consisted of 241 children with confirmed BM, children with compatible symptoms and signs of BM and a positive CSF Gram stain, latex agglutination, culture, or PCR or a positive blood culture, and of whom a filter paper blood sample was available. The median age was 15 months (interquartile range (IQR) 6, 43; range 1–161 months). Of these children, 47% (*n* = 114) were females and 53% (*n* = 127) males. The detailed information of the patients is shown in the Table 2, and numbers less than 241 refer to only those whose information was available. The diagnosis was made by the attending physician, and the diagnostic criteria have been described previously [38]. The control group consisted of 265 Angolan children with no history of BM, and with no active infection. The median age of the control group was 66 months (IQR 42, 114; range 0–186 months). The gender of the control group was registered in 191 cases; 37% (*n* = 71) were female and 63% (*n* = 120) were male. The control samples were collected after a Sunday service (*n* = 72), at other pediatric wards (*n* = 74), at the surgery ward (*n* = 18), at surgery outpatient visits (*n* = 59), or at vaccination visits (*n* = 42). The patients were enrolled from 2005 to 2008 and from 2012 to 2017, and the control group children were enrolled in 2008 and 2017.

### 2.2. Laboratory Tests, Clinical Findings, and Severity Factors of BM

Microscopic examination, leukocyte counts, and glucose, matrix metalloproteinase-8 (MMP-8), and protein concentration measurements were performed for CSF specimens taken at admission. CSF was cultured on blood and chocolate agar plates and bacteria were identified by Gram stain or standard bacteriological phenotypic methods. When available, a latex agglutination test (Pastorex Meningitis, Bio-Rad Laboratories Inc., Marne-La-Coquette, France) was conducted if >100 leukocytes/mm^3^ were present and bacterial culture was negative. Whenever possible, the remaining CSF sample was stored at –80 °C and shipped for PCR identification to the National Institute of Health, Lisbon, Portugal, or later to the Centre for Respiratory Diseases and Meningitis (CRDM), National Institute for Communicable Diseases (NICD), Johannesburg, South Africa [37]. Concentrations of MMP-8 were determined with a time-resolved immunofluorometric assay (Medix Biochemica, Espoo, Finland).

In a previous study in the Paediatric Hospital of Luanda, Pelkonen et al. identified prognostic factors for poor outcomes in BM [5]. In this study, we compared the genotype distributions of *TLR4* and *TLR9* between the previously identified prognostic factors for poor outcomes in BM. The studied laboratory variables were levels of glucose, protein, leukocytes, and MMP-8 in CSF at admission. In addition, the highest level of C-reactive protein (CRP) and leukocytes in blood were analyzed. Additionally, the following factors were assessed: Poor general condition, level of consciousness, convulsions, and Glasgow and Blantyre coma scores at admission. In addition, neurological sequelae (other than ataxia), deafness, blindness, other focuses of infection during hospital stay, and fatal outcomes were analyzed.

### 2.3. DNA Isolation

Blood samples for the genetic studies of the BM patients (*n* = 241) and the first set of controls (*n* = 74) were collected with a dried blood spot (DBP) collection card (PerkinElmer 226 Sample Collection Device, PerkinElmer, Waltham, MA, USA), as these patients had blood collected for other purposes as well. Later, the second control set of DNA samples (*n* = 191) were taken from healthy individuals with SK-1S DNA buccal swabs (Isohelix, Harrietsham, Kent, UK) to avoid pain in sampling.

Genomic DNA was extracted with the QIAamp^®^ mini DNA extraction kit (Qiagen, Hilden, Germany) according to the manufacturer’s protocol for extracted genomic DNA. DNA concentrations were determined by a spectrophotometer (NanoDrop 1000, Thermo Scientific, Waltham, MA, USA). The entire eluted DNA sample was stored at −20 °C for further examination.

### 2.4. Genotyping

*TLR4* 896A > G (rs4986790) and *TLR9*-1486T > C (rs187084) were determined by high-resolution melting analysis (HRMA). The analyses were performed using LightCycler 480 version 5.1 (Roche, Basel, Switzerland) with a SensiFAST HRMA melting master kit (Bioline, London, UK). The method for *TLR4* rs4986790 genotyping has been published in detail previously [39].

The primers for *TLR4* rs4986790 (forward 5′-ACCATTGAAGAATTCCGATTAGCA-3′, reverse 5′-CCAGGGAAAATGAAGAAACATTTG-3′) and for *TLR9* rs187084 SNP (forward 5′-TTATTCCCCTGCTGGAATGTCA-3′, reverse 5’-CTGTACTGGATCCTGGGGATG-3′) were designed with the Primer-BLAST design tool (National Center for Biotechnology Information (NCBI), U.S. National Library of Medicine, Bethesda, MD, USA) and were ordered from Sigma-Aldrich (Saint Louis, MO, USA). Amplicon sizes were 95 bp (*TLR4*) and 72 bp (*TLR9*).

In each reaction, the volume was 20 µL and consisted of 3 µL of genomic DNA (approximately 8 ng/µL) and 17 µL of master mix, including 10 µL of melting master dye and 0.2 µM of forward and reverse primers. The master mix provided a final concentration of 3 mM of MgCl_2_. The HRMA reactions were run at 95 °C for 10 min, followed by 44 cycles of amplification at 95 °C for 10 s, at 60 °C for 10 s, and at 72 °C for 15 s. After PCR, the final melting cycle conditions were outlined by LightCycler: First heating to 95 °C and holding for 1 min, followed by cooling to pre-hold temperature (40 °C). The melting interval for collecting fluorescence was set from 60 to 95 °C (a ramp rate of 0.02 °C/s). In each run, three DNA samples with known (sequenced) genotypes of *TLR4* (rs4986790) and *TLR9* rs187084 were used as controls.

Additionally, all samples with an atypical HRMA difference curve or melting peak were analyzed by Sanger sequencing in the Finnish Institute for Molecular Medicine (FIMM), Helsinki, Finland.

### 2.5. Statistical Analyses

Statistical analyses were performed using the JMP Pro software for Windows, version 14 (SAS Camous Drive, Cary NC, USA) and IBM SPSS Statistics for Windows, version 25 (IBM Corp., Armonk, NY, USA). Categorical variables are described using numbers (*n*) and percentages (%) and bivariate analyses were performed with Fisher’s exact test. Continuous data are described by using means and 95% confidence intervals (CIs) or medians and IQRs as appropriate. SNP data and continuous data were compared using a Mann–Whitney *U* test if the data were not normally distributed. Multivariate analysis (i.e., binominal logistic regression analysis) was performed for the clinical features if *p* < 0.05. Poor general condition, convulsions during admission, and weight below two standard deviations (SDs) were used as covariates. A *p*-value of <0.05 was considered statistically significant. A Hardy–Weinberg (HWE) test was used to calculate the observed genotype distribution in the control population. The differences in the numbers of patients are due to missing or non-determined data.

## 3. Results

### 3.1. Detection of Pathogens and Laboratory Values

**Figure 1:**
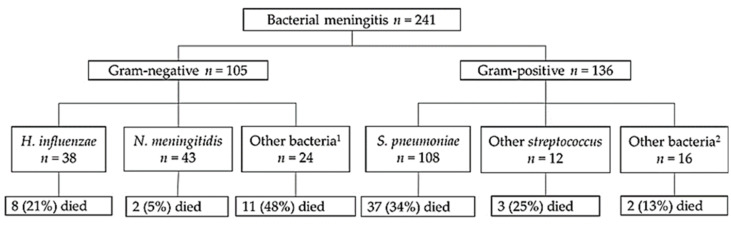
Distribution of the pathogens in the study population. ^1^ *Salmonella* spp. (*n* = 5), *Proteus* spp. (*n* = 6), *Citrobacter freundii* (*n* = 2), *Escherichia coli* (*n* = 2), *Klebsiella* spp. (*n* = 6), *Shigella* spp. (*n* = 1), *Enterobacter* spp. (*n* = 1), and unknown Gram-negative bacteria (*n* = 1). ^2^ *Staphylococcus aureus* (*n* = 3) and unknown Gram-positive bacteria (*n* = 13).

Bacterial etiology was confirmed from all 241 BM cases. Of these, 136 (56%) were caused by Gram-positive bacteria and 105 (44%) by Gram-negative bacteria. The bacterial distribution is presented in Figure 1. The three most common pathogens identified were *S. pneumoniae* (*n* = 108, 44.8%), *N. meningitidis* (*n* = 43, 17.8%), and *H. influenzae* (*n* = 38, 15.8%). The overall case fatality rate was 26% (*n* = 63). The highest fatality rate was 34% (*n* = 37) in pneumococcal meningitis, whereas the lowest fatality rate was 5% (*n* = 8) in meningococcal meningitis.

The laboratory findings of the BM patients are presented in Table 2. In brief, the median concentration of CRP was 161 mg/L and of leucocytes was 15.3/µL in the blood, while in the CSF, the concentration of glucose was 10 mg/dL, of protein was 201 mg/dL, of leucocytes was 1560/mm^3^, and of MMP-8 was 854 ng/mL.

### 3.2. Association between Susceptability to BM and the Gene Polymorphisms of TLR4 and TLR9

The frequencies of the studied *TLR4* and *TLR9* polymorphisms in the control group are presented in Table 3. Additionally, altogether, four subjects had another *TLR4* polymorphism in the area of interest in the *TLR4* gene. The frequency of the *TLR4* rs201050092 (902T > C) minor genotype (CT) was 0.01 in both groups. When the HWE was calculated for *TLR4* (rs4986790) and *TLR9* (rs187084), no deviations were found in the control group (*p* = 0.099 and *p* = 0.957) or in the patient group (*p* = 0.079 and *p* = 0.931).

The frequency of the variant genotypes AG and GG in *TLR4* was significantly higher in patients with *H. influenzae* meningitis than in the controls (OR, 2.5; 95% CI, 1.2–5.4; *p* = 0.021), whereas the frequency of the variant genotypes of CT and CC in *TLR9* was significantly lower in patients with *H. influenzae* meningitis than in the controls (OR, 0.4; 95% CI, 0.2–0.9; *p* = 0.036). No differences in the genotypes of *TLR4* and *TLR9* were found in patients with BM caused by other pathogens in comparison with the control children. The haplotype analyses between *TLR4* and *TLR9* did not show significant associations in patients with BM.

### 3.3. Association between Laboratory Values, Severity Factors of BM, and the TLR4 and TLR9 Polymorphisms

No associations were observed between the laboratory values or between the defined severity factors of BM and the studied SNPs across the entire study group. Table 4 shows the values from children who had meningitis caused by Gram-negative bacteria. The children who had meningitis caused by Gram-negative bacteria and who had a variant type of *TLR4* had significantly lower levels of MMP-8 (226 ng/mL (IQR, 64, 556) in their CFS at admission than those who had the AA genotype (1133 ng/mL (IQR, 834, 1642); *p* = 0.004). At the time of discharge, the children who carried a variant type of *TLR4* had a higher risk of ataxia (adjusted OR, 12.91; 95% Cl, 1.52–109.80; *p* = 0.019) and other neurological sequelae (adjusted OR, 11.85; 95% Cl, 1.07–131.49; *p* = 0.044).

In the Gram-positive BM group, children who were carriers of a variant type of *TLR4* (AG or GG) had lower levels of CSF glucose (7.5 mg/dL (IQR, 3.4, 12 03); *p* = 0.038) and blood leukocytes (9.5/µL (IQR, 5.06, 17.30); *p* = 0.024) at admission than those who had the AA genotype (Table S1).

A *TLR9* variant genotype seemed to play a different role to a *TLR4* variant type. In the bivariate analysis between the factors and *TLR9* polymorphisms, carriers of a *TLR9* variant genotype (CT or CC) had a lower risk of ataxia (OR, 0.22; 95% Cl, 0.06–0.84; *p* = 0.029) and other neurological sequelae (OR, 0.15; 95% Cl, 0.02–1.26; *p* = 0.049) at the time of discharge. After multivariate analysis, the differences were not statistically significant (Table 5).

In the Gram-positive BM group, no differences were observed between the studied *TLR9* SNPs, clinical features, and laboratory values (Table S2).

## 4. Discussion

In the present study, we analyzed the frequencies of the different genotypes of two functional SNPs of *TLR4* and *TLR9* in Angolan children with BM caused by different bacteria, and we compared this to controls matched for age and ethnicity. We observed a significant difference between patients with BM caused by *H. influenzae* and the controls. In addition, patients with meningitis caused by Gram-negative bacteria who were carriers of a variant *TLR4* genotype had an increased risk of ataxia and other neurological sequelae than those with a wild-type *TLR4* genotype. To the best of our knowledge, this is the first study to show that genetic variation in *TLR4* and *TLR9* is associated with *H. influenzae* meningitis in Angolan children.

*TLR4* SNP rs4986790 is one of the most studied SNPs and its variant genotypes AG and GG are associated with several infectious diseases. We recently reported that Finnish adults who are carriers of the variant genotypes of this *TLR4* SNP had significantly lower concentrations of serum IL-6, IL-12, and IL-17A than those who are carriers of the wild-type of *TLR4* [40]. It is known that *TLR4* variants are associated with hypo-responsiveness to LPS, and humans with these polymorphisms are more susceptible to infections caused by Gram-negative bacteria, and the disease is often more severe [10]. One study found a connection between *TLR4* SNPs and invasive pneumococcal sepsis [41]. Another study on survivors of meningococcal sepsis revealed an increased susceptibility between the *TLR4* rs4986790 heterozygous mutation and *N. meningitidis* infection [30,42]. However, other studies have shown contradictory results [43,44,45].

The effect of the *TLR9* SNP rs187084 has not yet been reported for pneumococcal or meningococcal infections, although it is associated with other bacterial infections. In puerperal women, an association between the *TLR9* polymorphism and *Streptococcus pyogenes* has been observed [46]. The *TLR9* polymorphism also has a functional significance in sepsis and multiple organ failure in patients with blunt trauma injury [47]. Furthermore, an association between persistent *Streptococcus aureus* nasal carriage and the *TLR9* polymorphism has been found [48].

In this study, we found a correlation between the *TLR4* and *TLR9* polymorphisms and *H. influenzae* meningitis in Angolan children. This finding suggests that genetic variations of *TLR4* rs4986790 and *TLR9* rs187084 play a role in the susceptibility to *H. influenzae* meningitis. Moreover, the variant genotypes AG and GG of *TLR4* rs4986790 are associated with increased susceptibility to *H. influenzae* meningitis. In contrast, the variant genotypes CT and CC of *TLR9* rs187084 seem to decrease the susceptibility to *H. influenzae* meningitis. We observed no associations between the analyzed *TLR4* and *TLR9* polymorphisms with other BM-causing pathogens, such as *S. pneumoniae* and *N. meningitidis*.

We also compared the allele frequencies of *TLR4* rs4986790 and *TLR9* rs187084 with African and European populations. In this study, the major A and minor G allele frequencies of *TLR4* rs4986790 in BM patients were 0.91 and 0.09, which are similar to those observed in the African population (A, 0.93; G, 0.07) and the European population (A, 0.94; G, 0.06) in the 1000 Genomes Project [49]. However, when comparing the genotype frequencies (AA, 0.68; AG, 0.26; GG, 0.05) in patients with *H. influenzae* meningitis to the African population (AA, 0.87; AG, 0.12; GG, 0.009) and the European population (AA, 0.89; AG, 0.10; GG, 0.006), significant differences were found. These differences were only found in patients with BM caused by *H. influenzae*, but not by *S. pneumoniae* or *N. meningitidis*. In addition, there was no such observation in the controls of this study [49]. The major T and minor G allele frequencies of *TLR9* rs187084 were 0.73 and 0.27 in BM patients, which are similar to the African population (T, 0.71; C, 0.29) but different from other populations, such as the European population (T, 0.57; C, 0.43). Although the genotype frequencies of *TLR9* in patients with BM caused by all pathogens are similar to that in the African population (TT, 0.514; CT, 0.387; CC, 0.098) in the 1000 Genomes Project, a significant difference was found in patients with *H. influenzae* meningitis (TT, 0.711; CT, 0.211; CC, 0.079) [50].

One of the most important diagnostic tools in BM is CSF analysis. A confirmed diagnosis of BM can be made when the bacterial culture of CSF is positive. Low glucose and elevated leukocyte count, protein levels, and MMP-8 and -9 in CSF can indicate severe BM [51,52,53]. However, in this study, we observed no association between the analyzed SNPs and the levels of glucose, protein, or leukocytes in the CSF, or of CRP and leukocytes in the blood. In a previous study in Latin America, our group showed that elevated MMP-8 levels in CSF predict poor disease outcomes in BM caused by *H. influenzae*, *N. meningitidis*, and *S. pneumoniae* [52]. In this study, we observed that the variant types of *TLR4* are associated with significantly lower levels of MMP-8 in children who had BM caused by Gram-negative bacteria, including *H. influenzae* and *N. meningitidis*. Since MMP-8 plays a central role in regulating tissue destruction, remodeling, and immune responses, this finding supports the broad role for the genetic variation of *TLR4* in clinical outcomes of children with BM caused by Gram-negative bacteria.

In this study, patients who had meningitis caused by Gram-negative bacteria and who were carriers of the variant *TLR4* genotypes had higher risk of ataxia and other neurological sequelae than those with the wild-type *TLR4* genotype. More association and functional studies on the polymorphisms of TLRs are needed to reveal the exact mechanism causing the differences in clinical course and to obtain genetic traits that can be used for patient profiling and management of patients with a CNS infection.

Identifying the risk factors for severe disease has been, historically, the first step toward helping patients. If the risk cannot be eliminated (such as age) or manipulated (such as undernutrition), the next step is to identify the mechanisms by which a disease causes harm. By understanding the underlying pathophysiology, one might then search for better treatment options. For instance, if a disease causes an exaggerated innate immune response, then this can be targeted in order to improve the course and outcome of the disease.

There are some limitations of our study. Although the total number of confirmed BM patients was relatively large, the number of individual causative pathogens such as *H. influenzae* and *N. meningitidis* was limited. Therefore, it is possible that we could have missed some weak associations. In this study, only one SNP of *TLR4* and *TLR9* was tested, and the possible effects of the other SNPs in the two genes on the susceptibility to and outcome of bacterial meningitis should also be kept in mind. In addition, the possible influence of genetic drift from the enrolled population on the results cannot be completely excluded.

## 5. Conclusions

The polymorphisms of *TLR4* rs4986790 (896A > G) and *TLR9* rs187084 (-1486T > C) were associated with susceptibility to *H. influenzae* meningitis in Angolan children. The variant genotypes of *TLR4* seemed to be associated with an increased risk of *H. influenzae* meningitis, whereas the variant genotypes of *TLR9* seemed to be associated with a decreased risk of *H. influenzae* meningitis. Furthermore, a variation in *TLR4* may increase the risk of the development of certain neurological sequelae after BM caused by Gram-negative bacteria. However, we observed no correlation between the analyzed SNPs and the other causative pathogens of BM. It should be noted that these results are valid only for children in Angola.

## Figures and Tables

**Table 1:** Functions of the analyzed single-nucleotide polymorphisms (SNPs) and allele frequencies in the African population.

Target	SNP ID	Location	Allele Frequency in the African Population	Allele Frequency in this Study	Potential Effect and Reported Associations
*TLR4*	rs4986790	Chromosome 9 In gene 896A > G	A: 0.93 G: 0.07	A: 0.91 G: 0.09	Diminished responsiveness: *M. catarrhalis* and *H. influenzae*, pneumococcal and meningococcal infections, septic shock, and respiratory syncytial virus (RSV)
*TLR9*	rs187084	Chromosome 3 Promoter area −1486T > C	T: 0.71 C: 0.29	T: 0.73 C: 0.27	Associated with autoimmune disorders and cancers, RSV, *Acinetobacter* *baumannii*, tuberculosis, and streptococcal infections

**Table 2:** Background, clinical, and laboratory data of patients.

Characteristics	Total Subjects ^1^	*N* (%)	Value Distribution	Range
Age in months, median (IQR)	241	-	15 (6, 43)	1–161
Male	241	127 (53%)	-	-
Weight (kg), median (IQR)	241	-	9 (6.8, 12.7)	3.6–36
**Clinical features**				
Fever (°C), mean (SD)	239	50 ^2^	37.5 (0.99)	35–40
Length of hospital stay in days, median (IQR) ^3^	158	-	12 (10, 36.05)	6–63
Poor general condition	238	142 (60%)	-	-
Convulsion in hospital	235	155 (66%)	-	-
Altered consciousness during admission	237	166 (70%)	-	-
Additional focus of infection present	232	69 (30%)	-	-
Dyspnea	238	125 (53%)	-	-
Glasgow coma score, median (IQR)	213	-	12 (9, 15)	3–15
Blantyre coma score, median (IQR)	212	-	4 (3, 5)	1–5
Fatal outcome	241	63 (26%)	-	-
Severe neurological sequelae at discharge	177	24 (14%)	-	-
**Laboratory tests, median (IQR)**				
CRP (mg/L)	207	-	161 (128, 161)	7–340
Glucose in CSF (mg/dL) ^4^	233	-	10 (5.5, 19.55)	0.5–270
Protein in CSF (mg/dL) ^4^	118	-	201.15 (136.2, 269)	10.2–1806
Leukocytes in CSF (/mm^3^) ^4^	241	-	1560 (414.5, 3590)	8–32,400
Blood leukocytes (/µL)	182	-	15.3 (10.2, 21.4)	1.23–57
MMP-8 in CSF (ng/mL)	92	-	854.2 (250, 1220)	3.74–3560

^1^ The total number of subjects was 241; numbers less than 241 refer only to those whose information was available. ^2^ Axillary temperature, fever if ≥38.0 °C. ^3^ The length of the hospital stay excludes patients with fatal outcome. ^4^ Measured at admission. IQR, interquartile range (lower, upper); SD, standard deviation; CRP, C-reactive protein; CSF, cerebrospinal fluid; MMP-8, matrix metalloproteinase-8.

**Table 3:** Genotype frequencies of the analyzed SNPs *TLR4* rs4986790 (AA compared to AG and GG) and *TLR9* rs187084 (TT compared to CT and CC) with the causative bacteria of bacterial meningitis (BM).

	Gram-Negative Bacteria			Gram-Positive Bacteria		
	*H. Influenzae*	*N. Meningitidis*	Other Bacteria	*S. Pneumoniae*	Other Bacteria	Control Group
*TLR4* rs4986790						
AA (%)	26 (68.4)	40 (93.0)	22 (91.6)	92 (85.2)	23 (82.1)	224 (84.5)
AG (%)	10 (26.3)	2 (4.6)	2 (8.3)	15 (13.9)	5 (17.9)	37 (13.9)
GG (%)	2 (5.3)	1 (2.3)	0	1 (0.9)	0	4 (1.5)
Total *n*	38	43	24	108	28	265
HWE	0.44	0.002	0.831	0.661	0.604	0.099
*p*-Value ^1^	0.022	0.164	0.549	0.999	0.784	Ref.
OR (95% CI)	2.5 (1.2–5.4)	0.4 (0.1–1.4)	0.5 (0.11–2.2)	1 (0.5–1.7)	1.2 (0.43–3.3)	
*TLR9* rs187084						
TT (%)	27 (71.1)	21 (50.0)	14 (58.3)	57 (55.3)	13 (46.4)	136 (51.7)
CT (%)	8 (21.1)	20 (47.6)	9 (37.5)	45 (43.7)	13 (46.4)	106 (40.3)
CC (%)	3 (7.9)	1 (2.4)	1 (4.2)	5 (4.9)	2 (7.1)	21 (8.0)
Total *n*	38	42	24	103	2	263
HWE	0.065	0.133	0.764	0.296	0.572	0.957
*p*-Value	0.036	0.869	0.670	0.819	0.692	Ref.
OR (95% CI)	0.4 (0.2–0.9)	1.1 (0.6–2.1)	0.76 (0.33–1.8)	0.9 (0.6–1.5)	1.2 (0.57–2.7)	

^1^ Bivariate analysis was calculated by Fisher exact test between BM and control group. OR, odds ratio; CI, confidence interval.

**Table 4:** Associations between the *TLR4* (rs4986790) polymorphism and the laboratory values and severity factors of Gram-negative bacterial meningitis (BM).

Clinical Features	*TLR 4*		OR (95% Cl)	*p*-Value
	AA	AG and GG		
Poor general condition ^1^	40 (46.0)	11 (64.7)	2.15 (0.73–6.35)	0.191
Convulsions during admission ^1^	32 (37.6)	8 (50.0)	1.66 (0.57–4.85)	0.409
Level of consciousness ^1^				
Normal	37 (43.5)	5 (29.4)	-	0.544
Altered	43 (50.6)	11 (64.7)	-	
Coma	5 (5.9)	1 (5.9)	-	
Glasgow coma score (<12) ^1^	31 (38.3)	9 (56.3)	2.07 (0.70–6.14)	0.266
Other focus of infection during hospital stay	49 (56.3)	10 (58.8)	1.11 (0.39–3.18)	0.999
Pneumonia during hospital stay	31 (35.2)	8 (47.1)	1.63 (0.57–4.66)	0.415
Outcome				
Fatal	15 (17.0)	6 (35.3)	2.66 (0.85–8.30)	0.102
Severe neurological sequelae ^3^	5 (6.9)	2 (18.2)	2.98 (0.50–17.68)	0.231
Deafness ^3^	5 (8.6)	0 (0.0)	0.91 (0.84–0.99)	0.999
Blindness ^3^	1 (1.4)	2 (20.0)	17.5 (1.42–215.21) - ^2^	0.039 (0.99) ^2^
Any neurological sequelae (no ataxia) ^3^	6 (8.3)	4 (40.0)	7.33 (1.31–33.41) 11.85 (1.07–131.49) ^2^	0.017 (0.044) ^2^
Ataxia ^3^	12 (16.9)	7 (70.0)	11.47 (2.59–50.80) 12.91 (1.52–109.80) ^2^	0.001 (0.019) ^2^
Laboratory variables, median (IQR)				
CRP (mg/L)	161.00 (131.5, 172.0) (*n* = 77)	160.00 (70.5, 161.0) (*n* = 13)	-	0.183
CSF glucose ^1^ (mg/dL)	10.40 (4.80, 22.10) (*n* = 88)	9.30 (5.15, 26.63) (*n* = 16)	-	0.786
CSF protein ^1^ (mg/dL)	191.85 (124.9, 257.15) (*n* = 46)	187.05 (146.48, 282.55) (*n* = 6)	-	0.748
CSF leukocytes ^1^ (/mm^3^)	1612.50 (970.00, 3830.50) (*n* = 88)	1440.00 (857.50, 1440.00) (*n* = 17)	-	0.748
Blood leukocytes (/µL)	16.11 (11.04, 24.35) (*n* = 73)	13.96 (8.23, 22.20) (*n* = 12)	-	0.434
CSF MMP-8 ^1^ (ng/mL)	1132.57 (834.04, 1641.75) (*n* = 30)	225.93 (63.56, 556.31) (*n* = 6)	-	0.004

^1^ Determined/measured at admission. ^2^ Determined at the time of discharge. ^3^ Adjusted OR; binominal logistic regression analysis. Poor general condition, convulsions during admission, and weight below 2 SD were used as covariates. IQR, interquartile range (lower, upper); CRP, C-reactive protein; CSF, cerebrospinal fluid; MMP-8, matrix metalloproteinase-8.

**Table 5:** Associations between the *TLR9* (rs187084) polymorphism and the laboratory values and severity factors of Gram-negative bacterial meningitis.

Clinical Features	*TLR9*		OR (95% Cl)	*p*-Value
	TT	CT and CC		
Poor general condition ^1^	28 (45.9)	23 (54.8)	1.43 (0.65–3.14)	0.426
Convulsions during admission ^1^	28 (46.7)	12 (30,0)	0.490 (0.21–1.14)	0.144
Level of consciousness ^1^				
Normal	25 (41.7)	17 (41.5)	-	0.928
Altered	31 (51.7)	22 (53.7)	-	
Coma	4 (6.7)	2 (4.9)	-	
Glasgow coma score (<12) ^1^	23 (41.1)	17 (42.5)	1.06 (0.47–2.41)	0.999
Other focus of infection during hospital stay	35 (57.4)	23 (54.8)	0.90 (0.41–1.99)	0.841
Pneumonia during hospital stay	24 (38.7)	15 (35.7)	0.88 (0.39–1.98)	0.838
Outcome				
Fatal	12 (19.4)	9 (21.4)	1.14 (0.43–3.00)	0.808
Severe neurological sequelae ^2^	7 (14.0)	0 (0.0)	0.86 (0.77–0.96) 0.00 (0.00–0.00) ^3^	0.039 0.999 ^2^
Deafness ^2^	3 (7.7)	2 (7.4)	0.96 (0.15–6.17)	0.999
Blindness ^2^	3 (3.1)	0 (0.0)	0.94 (0.87–1.01)	0.279
Any neurological sequelae (no ataxia) ^2^	9 (18.0)	1 (3.2)	0.15 (0.02–1.26) 0.17 (0.02–1.47) ^3^	0.049 (0.107)
Ataxia ^2^	16 (32.7)	3 (9.7)	0.22 (0.06–0.84) 0.25 (0.06–1.04) ^2^	0.029 (0.057) ^2^
Laboratory variables, median (IQR)				
CRP (mg/L)	161.00 (108.5,173.3) (*n* = 50)	161.00 (133.0, 161.0) (*n* = 39)	-	0.814
CSF glucose (mg/dL) ^1^	8.75 (4.73, 17.33) (*n* = 60)	16.40 (7.88, 25.53) (*n* = 38)	-	0.252
CSF protein (mg/dL) ^1^	189.10 (125.50, 275.00) (*n* = 31)	189.40 (131.15, 249.35) (*n* = 20)	-	0.549
CSF leukocytes (/mm^3^) ^1^	1518.50 (850.00, 3125.00) (*n* = 62)	1725.00 (990.00, 5607.50) (*n* = 42)	-	0.991
Blood leukocytes (/µL)	15.74 (10.89, 26.05) (*n* = 52)	15.63 (10.71, 21.14) (*n* = 32)	-	0.826
CSF MMP-8 (ng/mL) median	958.28 (319.46, 1496.89) (*n* = 20)	1187.47 (866.80, 1632.45) (*n* = 16)	-	0.278

^1^ Determined/measured at admission. ^2^ Determined at the time of discharge. ^3^ Adjusted OR; binominal logistic regression analysis. Poor general condition, convulsions during admission, and weight below 2 SD were used as covariates. IQR, interquartile range (lower, upper); CRP, C-reactive protein; CSF, cerebrospinal fluid; MMP-8, matrix metalloproteinase-8.

## Supplementary Materials

The following are available online at https://www.mdpi.com/2073-4425/11/9/1099/s1, Table S1: Associations between the *TLR4* polymorphism and the laboratory values and severity factors of Gram-positive bacterial meningitis; Table S2: Associations between the *TLR9* polymorphism and the laboratory values and severity factors of Gram-positive bacterial meningitis.
