# Supplementary material for: Gene Polymorphisms of TLR4 and TLR9 and Haemophilus influenzae Meningitis in Angolan Children
Source: Genes (Basel). 2020 Sep 21;11(9):1099. doi: 10.3390/genes11091099 (PMC7564843; doi:10.3390/genes11091099)
Supplement: Supplementary file 1 [file genes-11-01099-s001.pdf]

Table S1. : Associations between *TLR4* polymorphism (rs4986790) and laboratory values and severity factors of Gram-positive bacterial meningitis.

Clinical features	*TLR 4* AA	*TLR 4* AG and GG	OR (95% CI)	*p*-Value
Poor general condition[1]	75 (66.4)	16 (76.2)	1.62 (0.55–4.76)	0.453
Convulsions during admission[1]	61 (54.5)	13 (61.9)	1.36 (0.52–2.14)	0.635
Level of consciousness[1]				
Normal	24 (21.1)	5 (23.8)	-	
Altered	77 (67.5)	12 (57.1)	-	0.555
Coma	13 (11.4)	4 (19.0)	-	
Glasgow coma score (<12)[1]	50 (51.5)	11 (57.9)	1.29 (0.48–3.49)	0.401
Other focus of infection during hospital stay	62 (55.4)	11 (52.4)	0,89 (0.35–2.26)	0.816
Pneumonia during hospital stay	27 (23.5)	6 (28.6)	1.30 (0.4–3.69)	0.590
Outcome				
Fatal	33 (28.7)	9 (42.9)	1.86 (0.72–4.84)	0.208
Severe neurological sequelae[2]	15 (18.3)	2 (16.7)	0.89 (0.18–4.51)	0.999
Deafness[2]	11 (16.9)	0 (0.0)	0.83 (0.74–0.93)	0.341
Blindness[2]	11 (13.4)	0 (0.0)	0.87 (0.80–0.94)	0.350
Any neurological Sequelae (no ataxia)[2]	22 (28.2)	3 (25.0)	0.85 (0.21–3.43)	0.999
Ataxia[2]	28 (35.9)	3 (27.3)	0.67 (1.16– 2.73)	0.741
Laboratory variables, median (IQR)				
CRP (mg/L)	161.00 (125.25,; 161.00) (*n*=102)	161.00 (123.00,; 161.00) (*n*=15)	-	0.980
CSF-glucose[1] (mg/dL)	10.00 (6.30,; 19.53) (*n*=114)	7.5 (3.4,; 12.03) (*n*=20)	-	0.038
CSF-protein[1] (mg/dL)	225.70 (149.28,; 280.00) (*n*=56)	202.30 (174.40,; 245.60) (*n*=10)	-	0.761
CSF-leukocytes[1] (/mm^3)	1600.00 (280.00,; 3346.00) (*n*=115)	565.00 (221.00,; 4250.00) (*n*=21)	-	0.306
Blood leukocytes (/µL)	15.40 (10.23; 21.52) (*n*=84)	9.5 (5.06; 17.30) (*n*=13)	-	0.024
CSF-MMP-8[1] (ng/mL)	715.71 (183.71,; 922.00) (*n*=45)	574.28 (85.76; 1173.91) (*n*=10)	-	0.760

[1] Determined / measured at admission. [2] Determined at the time of discharge. OR, odds ratio; CI, confidence interval. IQR, interquartile range (lower, upper); CRP, C-reactive protein; CSF, cerebrospinal fluid; MMP-8, matrix metalloproteinase-8.

Formatted: Font: Italic

Table S2. : Associations between *TLR9* polymorphism (rs187084) and laboratory values and severity factors of Gram-positive bacterial meningitis.

Clinical features	*TLR9* TT	*TLR9* CT and CC	OR (95% Cl)	*p*-Value
Poor general condition[1]	47 (68.1)	43 (67.2)	0.96 (0.4 –1.98)	0.999
Convulsions during admission[1]	40 (57.1)	33 (53.2)	0.85 (0.43–1.70)	0.727
Level of consciousness[1]				
Normal	17 (24.6)	12 (18.5)	-	
Altered	41 (59.4)	47 (72.3)	-	0.269
Coma	11 (15.9)	6 (9.2)	-	
Glasgow coma score (<12)[1]	35 (59.3)	25 (44.6)	0.55 (0.26–1.16)	0.137
Other focus of infection during hospital stay	37 (53.6)	35 (55.6)	1.08 (0.54–2.15)	0.862
Pneumonia during hospital stay	37 (12.5)	23 (9.0)	0.74 (0.33–1.63)	0.549
Outcome				
Fatal	27 (38.6)	15 (23.1)	0.48 (0.23–1.01)	0.064
Severe neurological sequelae[2]	8 (18.6)	8 (16.0)	0.83 (0.28–2.45)	0.788
Deafness[2]	7 (20.0)	4 (10.3)	0.46 (0.12–1.72)	0.330
Blindness[2]	5 (11.6)	5 (10.2)	0.86 (0.23–3.21)	0.999
Any neurological Sequelae (no ataxia)[2]	12 (28.6)	12 (25.5)	0.86 (0.34–2.19)	0.813
Ataxia[2]	12 (28.6)	18 (39.1)	1.61 (0.66–3.93)	0.370
**Laboratory variables, median (IQR)**				
CRP (mg/L)	161.00 (117.5, –161.00) (*n*=61)	161.00 (134.0, –161.0) (*n*=55)	-	0.630
CSF-glucose[1] (mg/dL)	9.90 (5.83,; 19.90) (*n*=68)	10.20 (6.10,; 15.90) (*n*=65)	-	0.403
CSF-protein[1] (mg/dL)	196.50 (141.33,; 266.50) (*n*=32)	225.95 (160.03,; 287.66) (*n*=34)	-	0.514
CSF-leukocytes[1] (/mm^3)	1675.00 (298.75,; 3785.00) (*n*=70)	1200.00 (262.00,; 2800.00) (*n*=65)	-	0.770
Blood leukocytes (/µL)	14.90 (9.88,; 19.52) (*n*=45)	15.62 (10.52,; 21.90) (*n*=51)	-	0.997
CSF-MMP-8 (ng/mL)	721.17 (517.42,; 1220.40) (*n*=28)	384.79 (42.85,; 895.62) (*n*=26)	-	0.597

[1] Determined / measured at admission. [2] Determined at the time of discharge. OR, odds ratio; CI, confidence interval. IQR, interquartile range (lower, upper); CRP, C-reactive protein; CSF, cerebrospinal fluid; MMP-8, matrix metalloproteinase-8.
